# Integrated whole transcriptome profiling revealed a convoluted circular RNA-based competing endogenous RNAs regulatory network in colorectal cancer

**DOI:** 10.1038/s41598-023-50230-0

**Published:** 2024-01-02

**Authors:** Hasan Mollanoori, Yaser Ghelmani, Bita Hassani, Mohammadreza Dehghani

**Affiliations:** 1 Medical Genetics Research Center, Shahid Sadoughi University of Medical Sciences, Yazd, Iran; 2 Clinical Research Development Center, Shahid Sadoughi Hospital, Shahid Sadoughi University of Medical Sciences, Yazd, Iran; 3 Sarem Gynecology, Obstertrics and Infertility Research Center, Sarem Women’s Hospital, Iran University of Medical Sciences (IUMS), Tehran, Iran

**Keywords:** Gastrointestinal cancer, Tumour biomarkers, Data mining, Gene regulatory networks, Microarrays

## Abstract

Recently, it has been identified that circRNAs can act as miRNA sponge to regulate gene expression in various types of cancers, associating them with cancer initiation and progression. The present study aims to identify colorectal cancer-related circRNAs and the underpinning mechanisms of circRNA/miRNA/mRNA networks in the development and progress of Colorectal Cancer. Differentially expressed circRNAs, miRNAs, and mRNAs were identified in GEO microarray datasets using the Limma package of R. The analysis of differentially expressed circRNAs resulted in 23 upregulated and 31 downregulated circRNAs. CeRNAs networks were constructed by intersecting the results of predicted and experimentally validated databases, circbank and miRWalk, and by performing DEMs and DEGs analysis using Cytoscape. Next, functional enrichment analysis was performed for DEGs included in ceRNA networks. Followed by survival analysis, expression profile assessment using TCGA and GEO data, and ROC curve analysis we identified a ceRNA sub-networks that revealed the potential regulatory effect of hsa_circ_0001955 and hsa_circ_0071681 on survival-related genes, namely KLF4, MYC, CCNA2, RACGAP1, and CD44. Overall, we constructed a convoluted regulatory network and outlined its likely mechanisms of action in CRC, which may contribute to the development of more effective approaches for early diagnosis, prognosis, and treatment of CRC.

## Introduction

Colorectal cancer (CRC) accounts for 10 percent of all new cancers cases and 9.4 percent of cancer-related mortality around the world, ranking it third in terms of incidence and second in terms of mortality^1^. Despite significant advancements in understanding CRC, it was estimated that over 1.9 million new CRC cases and 935,000 deaths occurred in 2020, projected to rise to more than 2.2 million new cases and 1.1 million deaths by 2030^1,2^. Moreover, the mortality rate remains high, with an overall 5-year survival rate of only 50%, primarily due to late detection, high recurrence rates, and distant metastasis^3–5^. Detecting CRC at an early and localized stage significantly increases the 5-year survival rate to 90% and facilitates the identification and removal of adenomas, which can lead to colorectal cancer. So, developing methods for early detection is crucial in preventing colorectal cancer^6^.

Circular RNAs (circRNAs) belong to a newly discovered class of non-coding RNAs lack the 5′-end cap and 3′-end ploy A tail. They form covalently closed-continuous loop by linking the 5′ and 3′ ends generated through back splicing pre-mRNAs. This closed-loop structure confers circRNAs with resistance to exonucleases, making them more stable than linear transcripts^7,8^. Recent studies have demonstrated the different roles of circRNAs in cancer, including their involvement in cell proliferation, tumor metastasis, and drug resistance^9^. Among these roles, the most indicated function for circRNAs is acting as MicroRNA (miRNA) sponges. The competing endogenous RNA (ceRNA) hypothesis, first presented by Salmena et al., suggests that circRNAs efficiently sequester miRNAs, preventing their function and positively regulating the expression of their mRNA targets due to the shared miRNA response elements (MRE)^10^. Several studies have reported the dysregulation of circRNAs as a possible mechanism contributing to the development of CRC^11–14^. For instance, in CRC, overexpression of ciRS-7-a suppresses miR-7, thus alleviating the miR-7-mediated suppression of the EGFR/RAF1/MAPK pathway, a well-known oncogenic pathway that correlates with poor survival and metastasis^12^. Apart from their close association with cancer development, circRNAs' stability, conservation, and abundance in body fluids also make them valuable diagnostic biomarkers and potential therapeutic targets for cancer and other human diseases^15^.

In the present study, we utilized an integrated Systems Biology approach to construct circRNA-miRNA-mRNA ceRNA regulatory networks for CRC based on ceRNA hypothesis. We employed publicly available Gene Expression Omnibus (GEO) datasets for this purpose, and subsequently, we further assessed and refined our findings using data from the Cancer Genome Atlas (TCGA). To the best of our knowledge, this study represents the most comprehensive investigation providing potential circRNA-based ceRNA networks specific to CRC. The suggested ceRNAs regulatory networks derived from our study offer a novel perspective into the underlying molecular mechanisms driving CRC development, and they hold promise as innovative diagnostic/prognostic biomarkers and therapeutic targets.

## Results

### Identification of differentially expressed circRNAs, miRNAs, and genes

**Figure 1:**
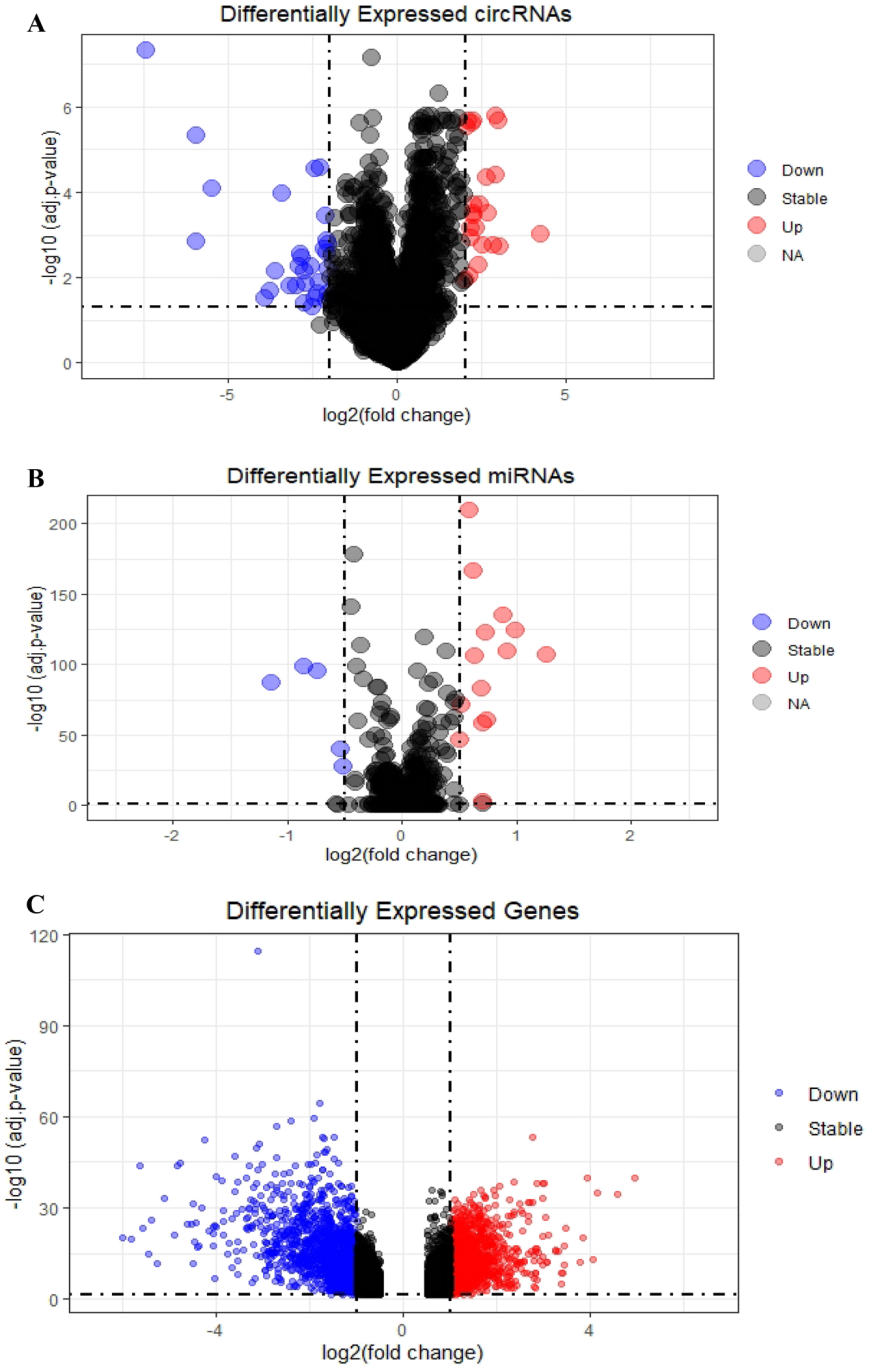
Volcano plots for (**A**) Differentially expressed circRNAs (DECs), (**B**) Differentially expressed miRNAs (DEMs), (**C**) Differentially expressed mRNAs (DEGs).

We consider circRNAs as a central element in constructing the competing endogenous RNA (ceRNA) network. The publicly available dataset GSE126094 provides expression profiles of 4012 circRNAs in 10 colorectal cancer (CRC) tissues and their corresponding normal-appearing tissues (NATs). Following hierarchical clustering, background correction, and normalization using limma, we conducted differential expression analysis with a cutoff value of an adjusted *p* value < 0.05 and a |log2 fold change (L2FC)|> 2.0 by limma. This analysis identified 54 significantly differentially expressed circRNAs (DECs), comprising 23 upregulated DECs and 31 downregulated DECs (Fig. 1A). These DECs exhibited distinct expression patterns in tumor tissues compared to normal tissues.

Similarly, for miRNA analysis in GSE115513, after hierarchical clustering, background correction, and normalization, we conducted differential expression analysis with a cutoff value of an adjusted *p* value < 0.05 and a |log2 fold change (L2FC)|> 0.5 using limma. This revealed 19 differentially expressed miRNAs (DEMs), with 14 upregulated DEMs and five downregulated DEMs (Fig. 1B). These miRNAs also showed distinct expression patterns in tumor tissues compared to normal tissues.

Furthermore, using GSE39582 as a discovery dataset, after hierarchical clustering, background correction, and normalization, we identified 2084 differentially expressed genes (DEGs) in tumor tissues compared to normal tissues using limma with a cutoff value of an adjusted *p* value < 0.05 and a |log2 fold change (L2FC)|> 1. Among these DEGs, 959 genes were upregulated, while 1125 genes were downregulated (Fig. 1C). For a comprehensive list of all differentially expressed circRNAs, miRNAs, and genes, please refer to Supplementary Tables 1–3.

### Identification of miRNA binding sites on DECs and their corresponding target miRNAs

Recent studies have underscored the potential role of circRNAs as ceRNAs in various malignancies, where they act by sequestering miRNAs. CircRNAs, containing MREs akin to those in miRNA target transcripts, competitively bind miRNAs, thereby regulating the expression of these target transcripts. To elucidate the interactions between circRNAs and miRNAs, we employed circBank, an online tool for predicting the presence of MREs on DECs. The intersection of DECs with the overlapping miRNAs, as predicted by circBank and DEMs analysis, led to the identification of eight circRNAs and nine miRNAs (Table 1).

**Table 1:** circRNA-miRNA interactions.

Selected DECs	Target miRNA
hsa_circ_0035445	hsa-miR-21-5p
hsa_circ_0071681	hsa-miR-29a-3p
hsa_circ_0065173	hsa-miR-19b-3p
hsa_circ_0000375	hsa-miR-663a
hsa_circ_0001955	hsa-miR-150-5p
hsa_circ_0092314	hsa-miR-17-5p and hsa-miR-93-5p
hsa_circ_0063331	hsa-miR-20a-5p and hsa-miR-93-5p
hsa_circ_0007158	hsa-miR-17-5p, hsa-miR-19b-3p, hsa-miR-20a-5p, hsa-miR-3651, and hsa-miR-93-5p

### Identification of miRNA–mRNA interaction

According to the ceRNA hypothesis, circRNAs efficiently sequester miRNAs, preventing their function and consequently positively regulating the expression of their mRNA targets through shared MRE. MiRNAs typically regulate the expression of downstream genes by interacting with the 3′ UTR of target mRNAs, thereby suppressing their expression. Utilizing miRWalk and combining the predicted mRNA targets of miRNAs with the DEGs, an overlapping analysis identified a total of 606 DEGs (Supplementary Table 4).

### GO enrichment analysis of selected DEGs

**Figure 2:**
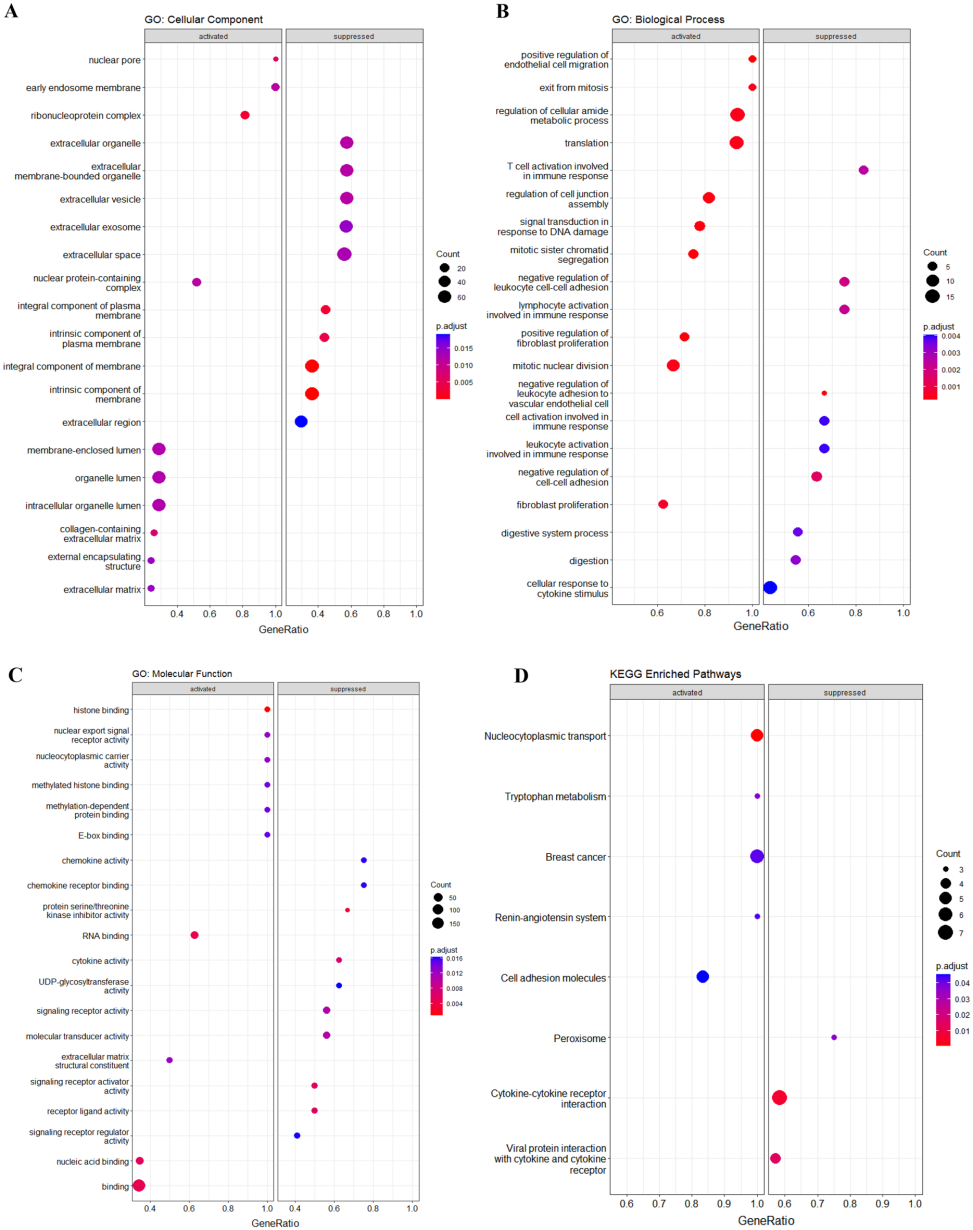
Functional enrichment analysis dot plots. (**A**) GO Cellular Component terms, (**B**) GO Biological Process terms, (**C**) GO Molecular Function terms, and (**D**) KEGG enriched pathways.

GO enrichment analysis was conducted on the selected DEGs, and the results are presented in Fig. 2. In terms of Cellular Component (CC) terms (Fig. 2A), the significantly enriched genes were primarily associated with organelle lumen, ribonucleoprotein complex, intrinsic/integral component of the membrane, extracellular exosomes/vesicle, and extracellular space/organelle. Regarding Biological Process (BP) terms (Fig. 2B), the enriched genes were mainly involved in the regulation of cellular amide metabolic processes, translation, mitotic nuclear division, regulation of cell junction assembly, negative regulation of cell–cell adhesion, and T cell activation in the immune response. And finally, for Molecular Function (MF) terms (Fig. 2C), the significantly enriched genes were predominantly associated with histone binding, binding of RNA/nucleic acids, protein serine/threonine kinase inhibitor activity, signaling receptor activator activity, receptor-ligand activity, and cytokine activity.

In summary, these results indicate that the selected genes play diverse roles in the tumor microenvironment (TME). They are involved in the composition of the extracellular matrix (ECM), a highly active part of the TME influencing tumor cell behavior and metastatic capability^16^. Additionally, they contribute to metabolic processes, cell division, and exhibit scaffold activity. Notably, the involvement of extracellular vesicles, significant signaling tools in the TME, further underscores the multifaceted roles these genes play in shaping TME.

### KEGG enrichment analysis of selected DEGs

Further analysis of KEGG pathways revealed the enrichment of eight pathways, as depicted in Fig. 2D. Activated pathways included nucleocytoplasmic transport, breast cancer, cell adhesion molecules, tryptophan metabolism, and the renin-angiotensin system. These findings not only enhance our understanding of CRC development but also open avenues for targeted therapeutic approaches.

The activated pathways suggest potential targets for therapeutic interventions. For instance, targeting nucleocytoplasmic transport^17^, employing Epithelial Cell Adhesion Molecule (EpCAM)-targeted treatments^18^, regulating tryptophan metabolism^19^, and manipulating the renin-angiotensin system are promising strategies^20^. Conversely, the pathways of cytokine-cytokine receptor interaction, viral protein interaction with cytokine and cytokine receptors, and peroxisome were found to be suppressed.

Understanding the role of cytokines in TME is crucial, as cancer cells communicate with their niche via cytokines to regulate various aspects of tumor development, growth, invasion, metastasis, and therapy resistance. Thus, cytokine-mediated therapy emerges as a promising avenue for novel treatment strategies in CRC^21^. Additionally, targeting peroxisome metabolism, implicated in the WNT/β-catenin pathway and fatty acid oxidation, holds promise as a novel therapeutic strategy for CRC treatment, particularly in the context of high-fat diet-induced obesity^22^.

In conclusion, the identification of these enriched pathways not only deepens our understanding of CRC pathogenesis but also provides valuable insights for the development of targeted therapeutic strategies, ranging from nucleocytoplasmic transport to cytokine-mediated therapy and peroxisome metabolism.

### Construction of circRNAs/miRNAs/mRNAs regulatory network

**Figure 3:**
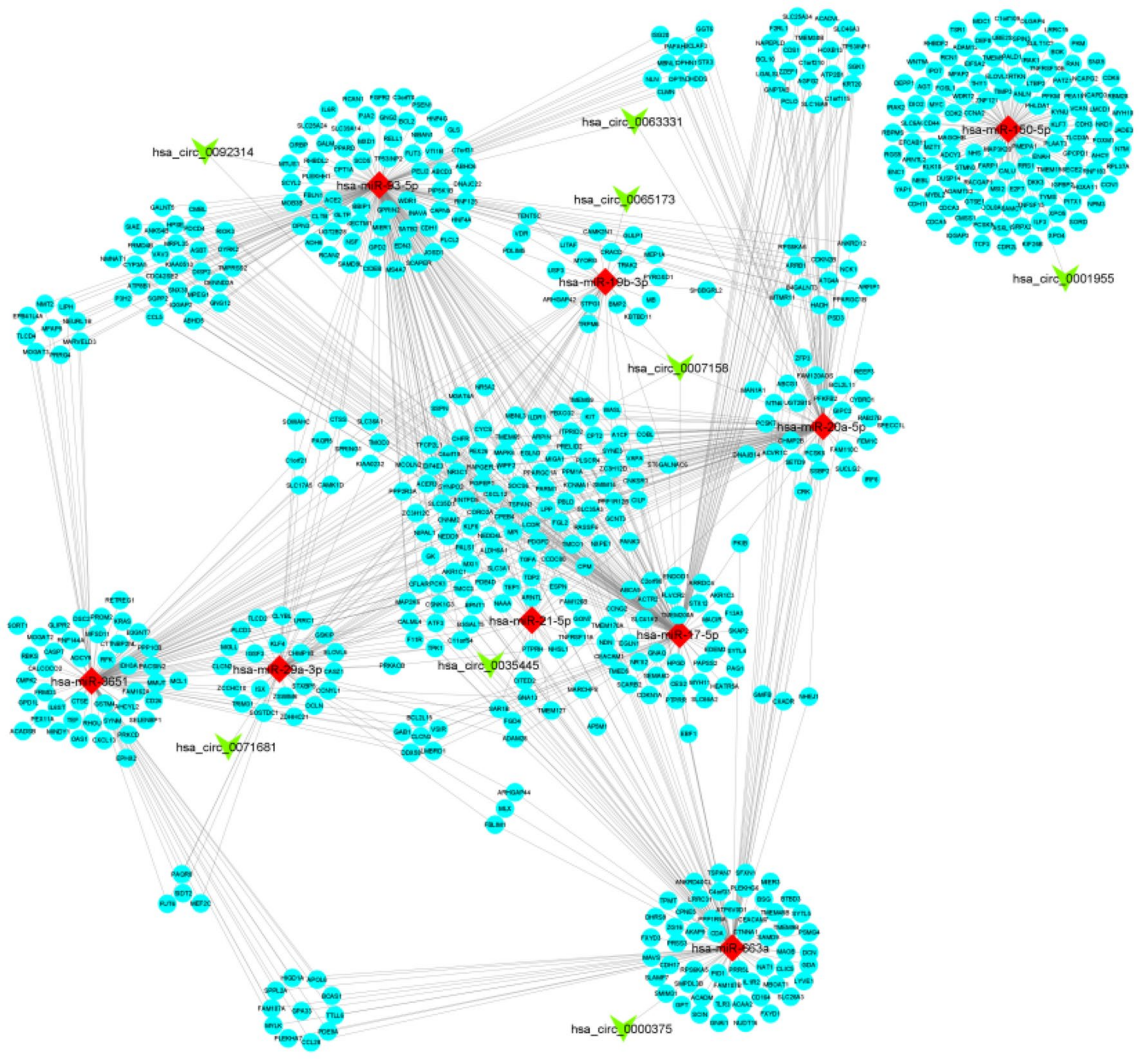
ceRNA regulatory network. Ellipse represents Gene, V represents circRNA, and Diamond represents miRNA.

The complete ceRNA regulatory network, integrating the DECs/DEMs pairs and DEMs/DEGs pairs, is visually represented in Fig. 3. Utilizing Cytoscape software, we constructed a holistic interaction network among the DECs/DEMs pairs and DEMs/DEGs pairs identified in the previous steps. Within this network, circRNAs, miRNAs, and mRNAs serve as nodes, representing their respective roles, and the edges signify the interactions between these components.

Adhering to the ceRNA hypothesis, circRNAs, acting as miRNA sponges, negatively regulate miRNAs and consequently positively regulate their mRNA targets. The ceRNA network encompasses eight circRNAs, nine miRNAs, and 606 genes, offering a comprehensive overview of the interconnected relationships among circRNAs, miRNAs, and mRNAs in colorectal tumors. This network elucidates the functional connections between mRNAs and non-coding RNAs, revealing potential post-transcriptional regulatory effects among them.

The presented ceRNA network provides a thorough perspective on the molecular mechanisms driving CRC development. Moreover, it holds promise as a source of innovative diagnostic/prognostic biomarkers and therapeutic targets in CRC.

### Construction of protein–protein interactions (PPIs) network and detection of hub genes

**Figure 4:**
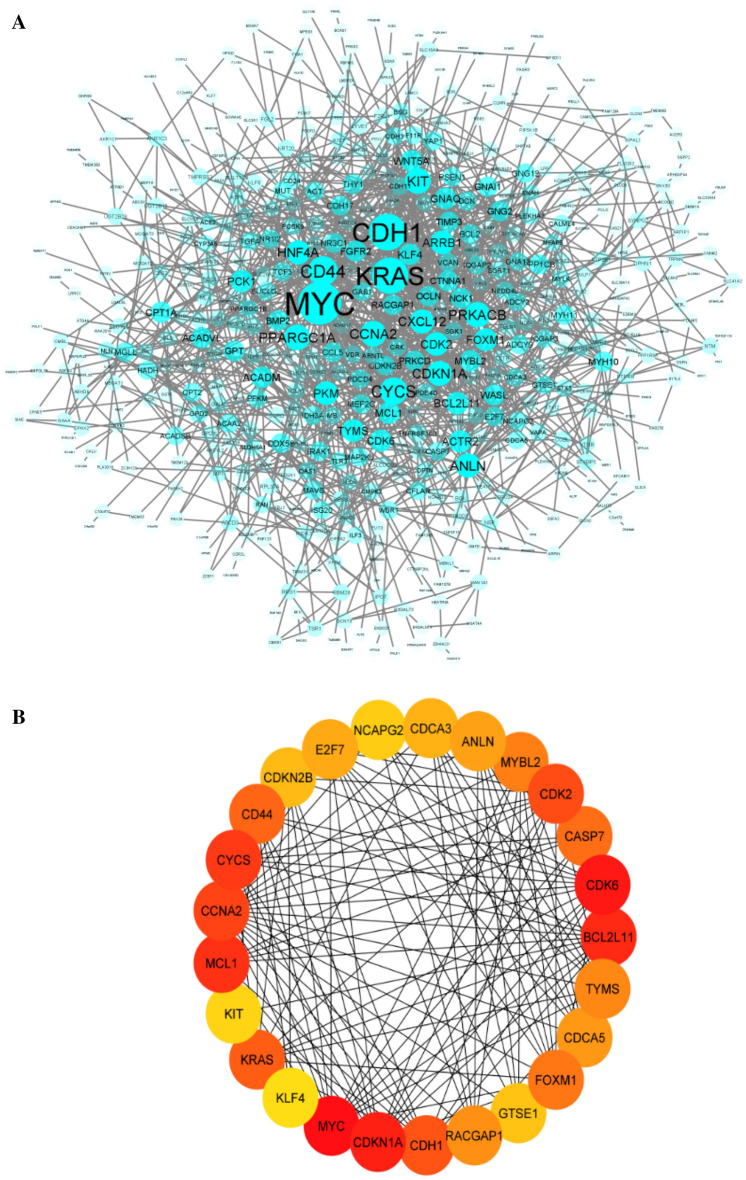
(**A**) The protein–protein interaction (PPI) network. The size and transparency of the node reflects the degree value of the genes, which depends on the number of their interaction. Nodes with more interactions are larger and less transparent. (**B**) The network of 20 hub genes of the PPI network by applying the MCC ranking score. The significance of Hub Genes reflects on a color scale ranging from red to yellow. The importance of hubs decreases from red to yellow.

Cells actively engage in a critical and intricate network of functional associations involving various biomolecules within the body. Among these interactions, Protein–Protein Interactions (PPIs) hold particular significance due to their versatility, specificity, and adaptability. The PPI network, visualized using Cytoscape after removing unconnected proteins, is composed of 509 nodes representing interconnected proteins and 1557 edges that illustrate the interactions among them (Fig. 4A).

Within this extensive network, certain genes emerge as particularly important—these are referred to as hub genes. Hub genes, characterized by a high number of interactions with other genes, typically play essential roles in gene regulation and various biological processes^23^. To identify such hub genes, we employed the CytoHubba plugin, resulting in the identification of the top 25 hub genes. These genes are detailed in Fig. 4B and provided in Supplementary Table 5.

### Prognostic analysis of hub genes using Kaplan–Meier plots

**Figure 5:**
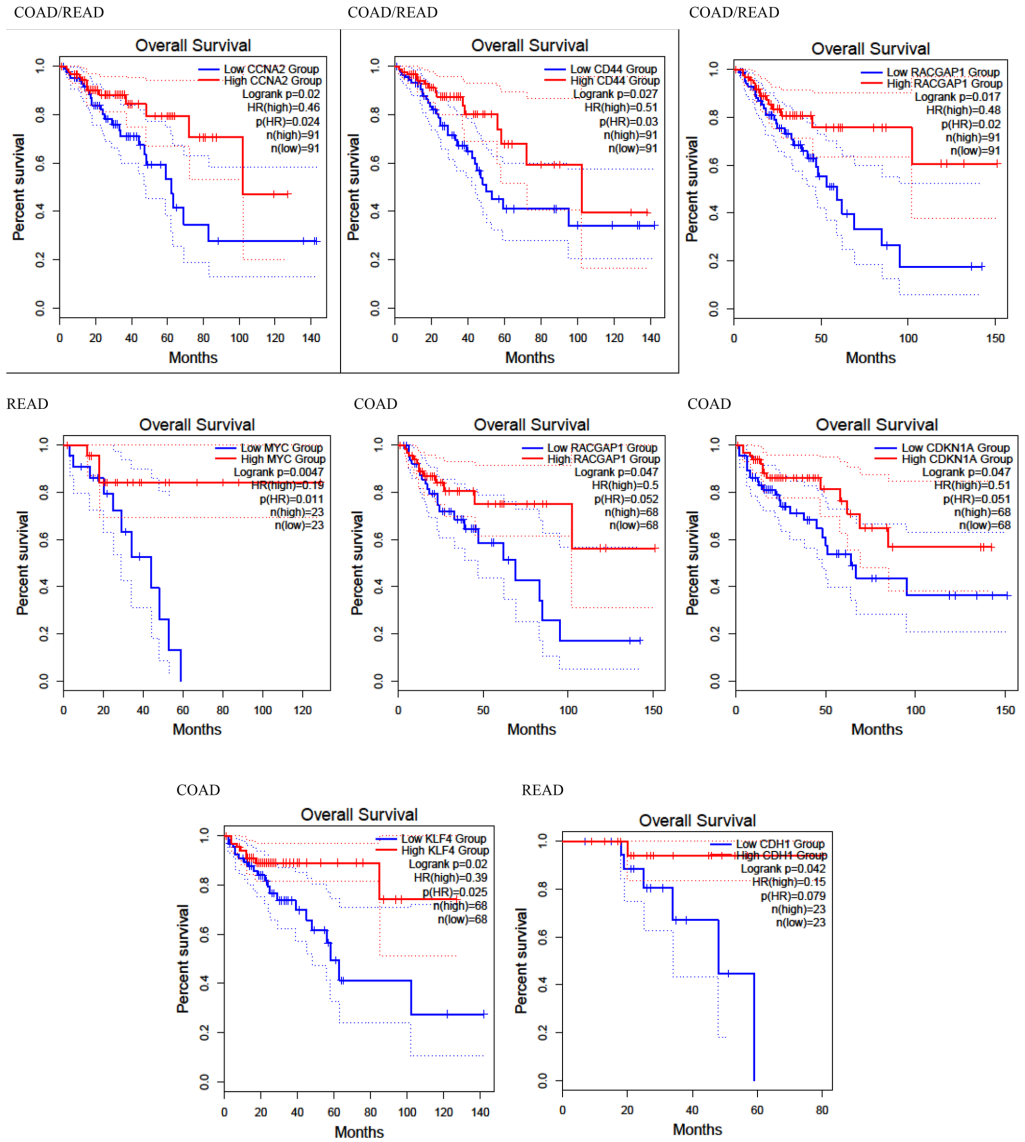
The Kaplan–Meier curve plots of CCNA2, CD44, RACGAP1, MYC, CDKN1A, CDH1, and KLF4 for the overall survival of CRC patients based on TCGA data extracted from GEPIA database.

The prognosis analyses of 25 hub genes were conducted using the GEPIA online tool, utilizing expression data from 68 colon cancer patients and 23 rectal cancer patients. In Fig. 5, Kaplan–Meier plots illustrate the survival analysis for seven statistically significant hub genes: CCNA2 (log rank *p* value: 0.02), CD44 (log rank *p* value: 0.027), RACGAP1 (log rank *p* values: 0.017 and 0.047), MYC (log rank *p* value: 0.0047), CDKN1A (log rank *p* value: 0.047), CDH1 (log rank *p* value: 0.042), and KLF4 (log rank *p* value: 0.02).

These genes exert a notable impact on the overall survival of patients with colon adenocarcinoma (COAD) and rectal adenocarcinoma (READ). An interpretation based on the Kaplan–Meier plots reveals that MYC significantly affects prognosis in READ patients, while CD44 has a slight effect on prognosis for COAD patients.

### Assessment of survival-related hub genes expression profiles by validation datasets and TCGA data

To assess the reproducibility of the DEGs analysis, we meticulously searched the GEO repository for datasets similar to GSE39582. After a comprehensive review, we selected four datasets (GSE44076, GSE8671, GSE9348, and GSE21510) that, like GSE39582, utilize the Affymetrix platform and compare cancer tissue against adjacent normal tissues. All genes identified as survival-related were confirmed to be differentially expressed in these validation datasets.

Consistency in the differential expression status, whether upregulated or downregulated, was observed across all datasets for CCNA2 (adjusted *p* values: 1.54E−25, 4.93E−09, 1.22E−17, and 2.48E−28 for GSE44076, GSE9348, GSE8671, and GSE21510, respectively), CD44 (adjusted *p* values: 5.22E−70, 1.83E−09, 1.21E−32, and 1.43E−39, respectively), MYC (adjusted *p* values: 9.84E−77, 9.93E−19, 2.02E−27, and 3.2E−38, respectively), RACGAP1 (adjusted *p* values: 5.17E−43, 3.99E−-13, 1.48E−17, and 1.65E−30, respectively), and KLF4 (adjusted *p* values: 2.93E−74, 4.41E−16, 2.44E−20, and 1.57E−53, respectively). CDH1 was differentially expressed only in GSE9348, and CDKN1A showed differential expression in GSE9348, GSE44076, and GSE21510 but not in GSE8671 (Supplementary Tables 6–9).

**Figure 6:**
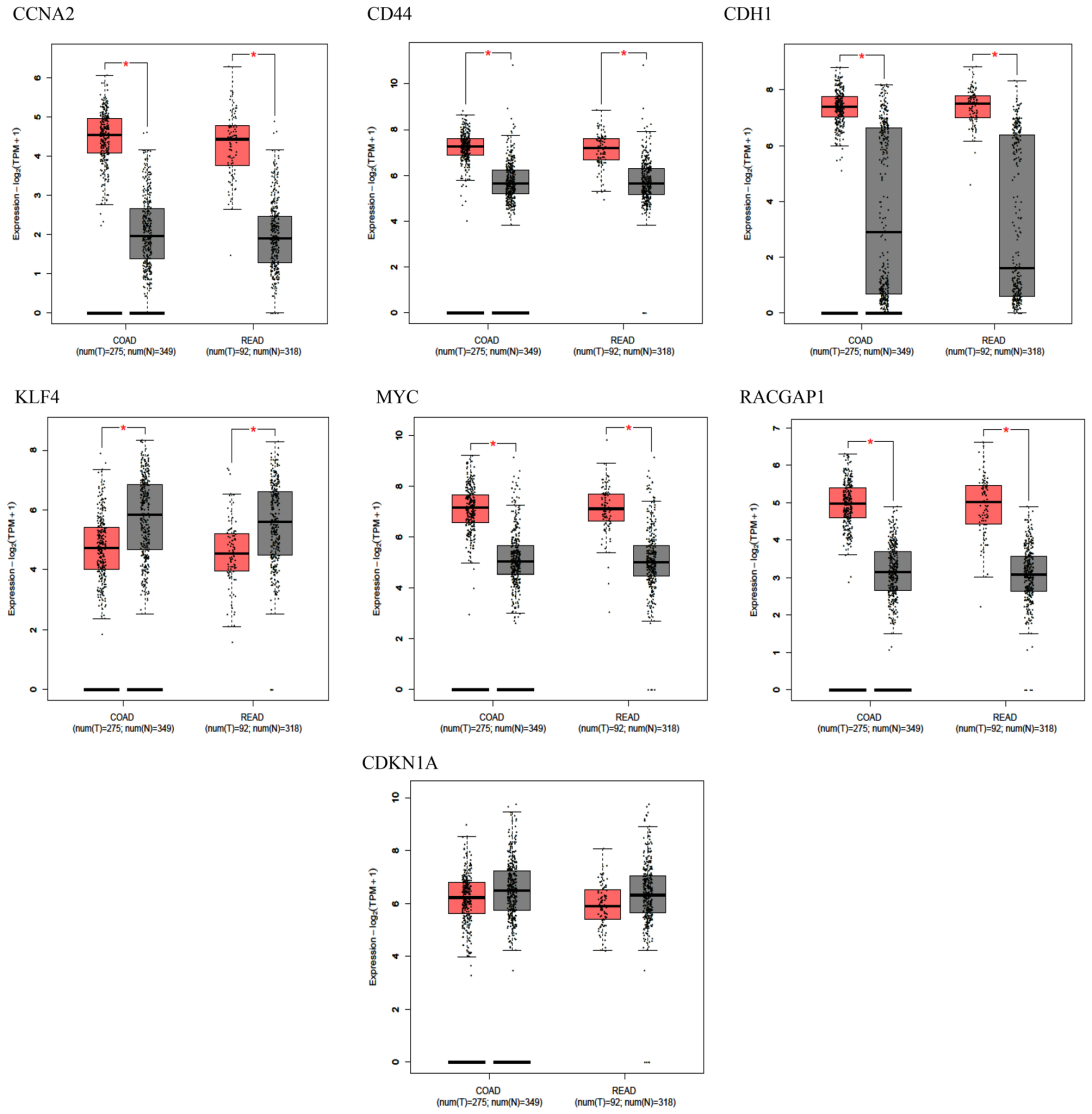
Expression status of survival related Hub Genes based on TCGA data extracted from GEPIA database. Tumors and normal samples present in red and gray, respectively. Red star indicates significant difference between tumors and adjacent normal tissues (*p* value ≤ 0.05).

Additionally, results from GEPIA, as depicted in Fig. 6, confirmed the expression profiles of CCNA2, CD44, MYC, RACGAP1, and KLF4, aligning with the results obtained from the DEGs analysis in GSE39582. These consistent results across multiple datasets, including both microarray and RNAseq analyses, underscore the reproducibility of the expression profiles of CCNA2, CD44, MYC, RACGAP1, and KLF4 CRC. As a result, these genes were selected for further sensitivity and specificity assessment.

### Receiver operating characteristic (ROC) curve analysis and constructing a ceRNA sub-network

**Figure 7:**
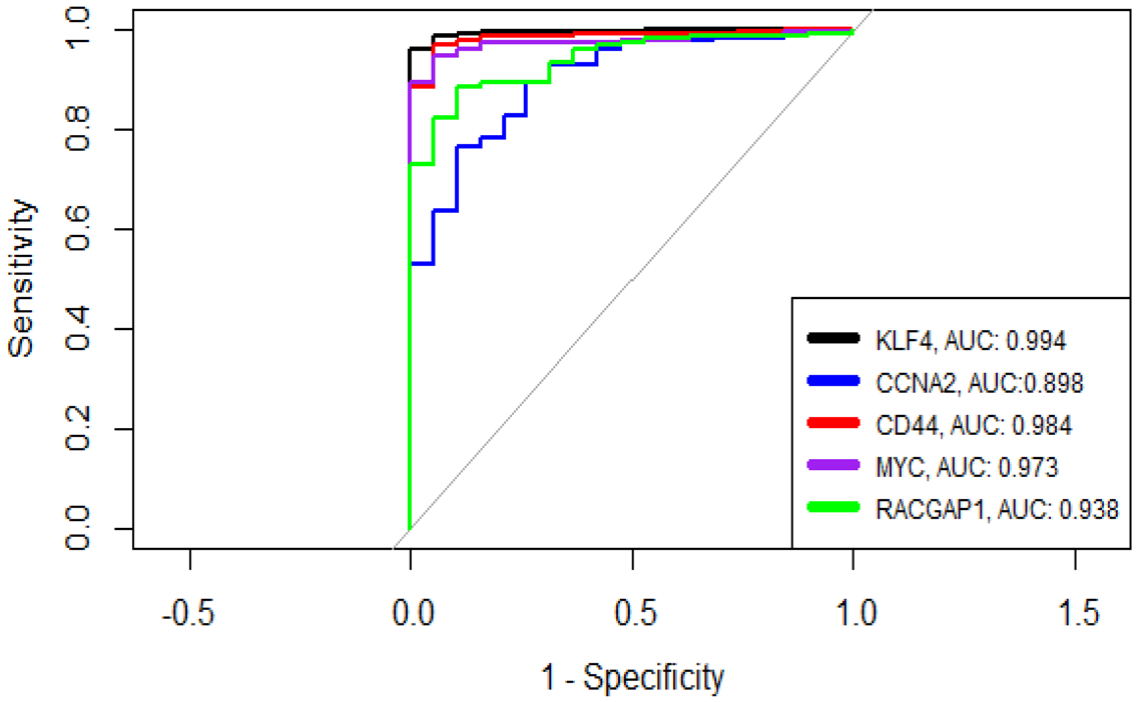
ROC curves for validated survival-related Hub Genes.

All selected genes underwent validation using both validation datasets and TCGA data, demonstrating consistently high Area Under the ROC Curve (AUC) scores. Specifically, KLF4, CCNA2, CD44, MYC, and RACGAP1 exhibited AUC scores of 0.994, 0.898, 0.984, 0.973, and 0.938, respectively (as illustrated in Fig. 7). These high AUC scores attest to the accuracy and reliability of these genes as diagnostic and prognostic biomarkers in CRC.

**Figure 8:**
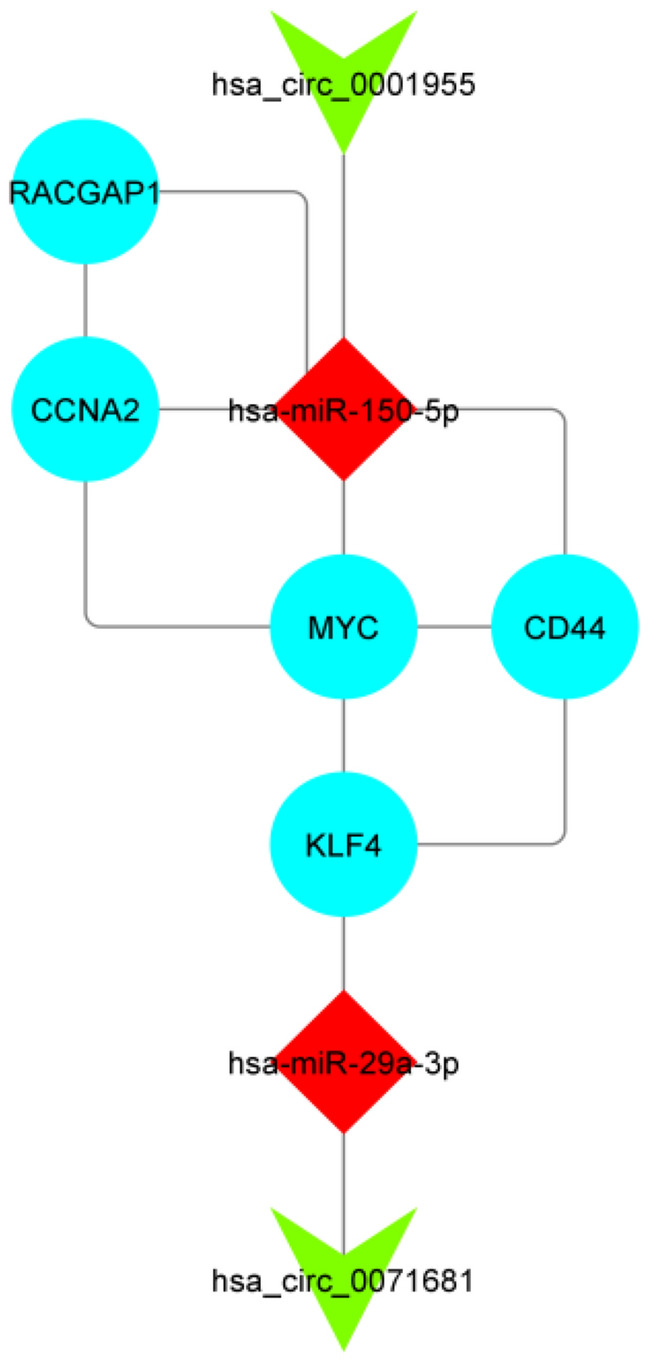
ceRNA regulatory subnetwork. Ellipse represents Gene, V represents circRNA, and Diamond represents miRNA. The size of the node reflects the degree value of the genes, which depends on the number of their interaction.

To streamline the comprehensive ceRNA regulatory network and focus on hub genes relevant to survival, reproducibility, and accuracy, we constructed a ceRNA sub-network. This sub-network comprises two circRNAs, two miRNAs, and five hub genes, as depicted in Fig. 8. Overall, these findings contribute to a deeper understanding of the underlying mechanisms in the development and prognosis of CRC. Moreover, they pave the way for the development of novel therapeutic approaches based on this regulatory network.

## Discussion

In recent decades, considerable advancements in technology and clinical trials have significantly improved the detection, staging, and treatment of CRC. However, despite these developments, CRC remains one of the leading causes of cancer-related deaths worldwide^24,25^. One of the significant reasons for its high mortality rate and poor overall survival is late detection^5^. While CRC survival rate are high when detected early (up to 90 percent five-year survival for early detection at stage 1), it significantly decreases for late diagnosis. As a result, researchers have made many attempts to enable early-stage diagnosis of CRC. Up to now, the application of molecular biomarkers in the detection of CRC is limited to carcinoembryonic antigen (CEA) as a serum biomarker for monitoring disease recurrence or response to treatment and KRAS to detect resistance to the anti-EFGR chemotherapy drug Cetuximab^26^.

CircRNAs, as one of these RNA types, act as potential regulatory components. Several studies have demonstrated that circRNAs are expressed differentially in tumor tissues compared to normal tissues and have been implicated in CRC pathogenesis, as comprehensively reviewed in the paper by Long et al.^27^. However, our knowledge about circRNA-associated ceRNA networks in CRC is still insufficient, and there may be plenty of circRNAs that require further study. In the current study, we developed a series of in-silico analyses to identify a comprehensive circRNA-associated ceRNA network in CRC pathogenesis.

After conducting a differential expression analysis on GSE126094, we identified 54 circRNAs as DECs. Subsequently, through the intersection of miRNAs obtained from the circBank database and DEMs extracted from the differential expression analysis of GSE115513, we pinpointed eight DECs that have the potential to act as sponges for nine corresponding miRNAs, thus negatively regulating their expression. The identified circRNA-miRNA pairs include hsa_circ_0035445/hsa-miR-21-5p, hsa_circ_0071681/hsa-miR-29a-3p, hsa_circ_0065173/hsa-miR-19b-3p, hsa_circ_0000375/hsa-miR-663a, hsa_circ_0001955/hsa-miR-150-5p, hsa_circ_0092314/hsa-miR-17-5p/hsa-miR-93-5p, hsa_circ_0063331/hsa-miR-20a-5p/hsa-miR-93-5p, and finally hsa_circ_0007158/hsa-miR-17-5p/hsa-miR-19b-3p/hsa-miR-20a-5p/hsa-miR-3651/hsa-miR-93-5p.

By intersecting mRNAs obtained from the miRWalk database with the DEGs identified in the GSE39582 dataset through a differential expression analysis, we adhered to the ceRNA hypothesis and identified 606 genes. Using this information, a comprehensive ceRNA network was constructed, consisting of eight circRNAs, nine miRNAs, and 606 genes. The DEGs analysis employed a cut-off value of an adjusted *p* value of < 0.05 and a |log2 fold change (L2FC)|> 2.0, while the DEM analysis used a cut-off value of an adjusted *p* value of < 0.05 and a |log2 fold change (L2FC)|> 0.5. Similarly, the DEGs analysis applied a cut-off value of an adjusted *p* value of < 0.05 and a |log2 fold change (L2FC)|> 1. This network provides valuable insights into the intricate connections among circRNAs, miRNAs, and mRNAs in the development of CRC.

Further analysis involved survival analysis, expression profile assessment using TCGA and GEO datasets (with an adjusted *p* value of < 0.05 and a |log2 fold change (L2FC)|> 1), and ROC curve analysis (AUC > 0.85). Subsequently, we identified five mRNAs for constructing a sub-network. This sub-network included hsa_circ_0001955, hsa_circ_0071681, hsa-miR-29a-3p, hsa-miR-150-5p, KLF4, MYC, CCNA2, RACGAP1, and CD44, provides a focused and meaningful representation of the selected genes and their interactions in CRC.

The role of hsa_circ_0001955 in CRC has been extensively studied, revealing its involvement in progression, apoptosis, and cell proliferation. In a 2019 study, Chen et al. demonstrated that hsa_circ_0001955 might function as a sponge of hsa-miR-597-5p to upregulate CDK6 and RPA3 expression, promoting CRC progression^28^. Their results showed that in vitro and in vivo silencing of hsa_circ_0001955 expression induced apoptosis, repressed cell proliferation, and impaired the DNA repair capacity of CRC cells. Additionally, other studies have revealed that upregulation of hsa_circ_0001955 may contribute to the pathogenesis and progression of CRC^29–31^. Based on our literature review, there were no reports of the involvement of the has_circ_0071681 in CRC.

The two miRNAs contributing to the sub-network play integral roles in various biological processes associated with development, metastasis, and therapy resistance in CRC. Upregulation of hsa-miR-29a-3p has been observed in the tissue and plasma of CRC patients in several studies^32,33^. The involvement of hsa-miR-29a-3p has been indicated in various aspects of CRC, including angiogenesis, radiosensitivity, EMT, cell proliferation, apoptosis, metastasis, and drug resistance^34–38^. Furthermore, Wang et al.’s study revealed that hsa-mir-29a-3p could induce PI3K/Akt signaling pathway by negatively regulating PTEN expression^36^. Likewise, several functions have been described for hsa-miR-150-5p in CRC development, such as tumorigenesis, progression, tumor proliferation, angiogenesis, and metastasis^39–43^. Importantly, hsa-miR-150-5p directly target β-catenin and suppresses CRC progression^44^ or represses HMGA2, leading to the downregulation of Cyclin A and inhibition of CRC proliferation^45^. Additionally, researchers have suggested its role in the regulation of Akt/mTOR and Wnt/β-catenin pathways^40,41,44^.

### KLF4

Krüppel-like factor 4 (KLF4), a conserved zinc finger-containing transcription factor, is implicated in various aspects of CRC, including proliferation, metastasis, and genome instability. The hsa_circ_0071681/hsa-miR-29a-3p/KLF4 axis may play crucial role in CRC cell proliferation. KLF4 is known to be downregulated in CRC compared to normal tissues and acts as a tumor suppressor^46^. Fu et al.’s study revealed that hsa-mir-29a-3p targets KLF4 and negatively regulates KLF4 mRNA and protein expression^47^. Additinally, the knockdown of KLF4 in the SW480 CRC cell line induced the YAP pathway and its downstream genes, including EGFR, MYC, BIRC5, and CTGF, leading to tumor proliferation^48^. On the other hand, downregulation of KLF4 can induce β-catenin expression and activate Wnt/β-catenin signaling pathway^49^. The activation of Wnt/β-catenin signaling, in turn, activates the YAP signaling pathway, leading to unlimited cell proliferation in response to DNA damage, mechanical stress, or inflammatory signals^50^. Thus, the downregulation of KLF4 may play a role in tumor proliferation by inducing β-catenin expression and activating Wnt/β-catenin signaling pathway. The DEGs analysis in our present study showed upregulation of YAP1, MYC, and BIRC5 in CRC tissues, which supports our suggested theory.

The axis may also impact CRC cell proliferation by affecting the expression of NDRG2 and cyclin D1 (CCND1)^51–53^. Downregulation of NDRG2 and upregulation of CCND1 in CRC tissues compared to normal tissues in our DEG analysis further support this notion. Additionally, KLF4 downregulation may promote stem cell-like properties of CRC cells by influencing Wnt/β-catenin pathway activity, which is characteristic of CRC^54–56^.

Moreover, the hsa_circ_0071681/hsa-miR-29a-3p/KLF4 axis could be involved in CRC invasion and metastasis. Hsa-miR-29a-3p has been shown to directly suppress KLF4 to regulate MMP2 and E-cadherin expression, promoting CRC metastasis^38^. This axis may also contribute to colon cancer progression and metastasis by inducing IFITM3 expression (directly by inhibition of IFITM3 transcription or indirectly by activating the Wnt/β-catenin pathway) and regulating EMT in CRC by targeting Snail and E-cadherin^53,57^.

Furtheremore, KLF4 is crucial for maintaining genomic stability through repressing cyclin E (CDK2) expression, preventing centrosome amplification following DNA damage^58^. Recently, Yang et al. revealed that intestinal epithelium-specific deletion of Klf4 causes increased genetic instability in colitis-associated colorectal cancer (CAC) in a mice model^46^. These findings suggested a guarding role for KLF4 against genetic instability in CRC. The observed elevation of CDK2 in DEGs analysis, which negatively correlates with KLF4 expression, strengthens the potential involvement of the hsa_circ_0071681/hsa-miR-29a-3p/KLF4 axis in genome stability.

Overall, the hsa_circ_0071681/hsa-miR-29a-3p/KLF4 axis appears to be a critical player in CRC development, influencing cell proliferation, stem cell-like properties, invasion, metastasis, and genomic stability.

### MYC

MYC is a family of regulator genes encoding transcription factors that function as proto-oncogenes. In CRC, MYC upregulation is a common characteristic in approximately 70% of cases. This upregulation can occur at the transcriptional level through elevated β-catenin levels and at the post-translational level through phosphorylation at serine 62, mediated by ERK^59^. c-MYC plays a key role in CRC tumorigenesis, apoptosis, and glycolytic metabolism, acting as a downstream effector of cancer stems cell (CSC) related signaling pathways like Wnt/β-catenin, Hedgehog, and Notch pathways involved in CSCs regulation in CRC^60–63^. Interestingly, MYC, as a master transcription factor, regulates over 15% of the human transcriptome, but there is little overlap between MYC-bound and MYC-regulated genes. Psathas et al. proposed a post-transcriptional model where MYC dysregulates specific miRNAs that target a broad spectrum of genes. Recent studies have shown that MYC can induce the hsa-miR-17–92 cluster (including, hsa-miR-17, hsa-miR-18a, hsa-miR-19a, hsa-miR-20a, hsa-miR-19b, and hsa-miR-92a) and suppresses hsa-miR-150-5p in B cell lymphoma^64,65^. Based on this evidence and our analyses, it’s likely that MYC is indirectly overexpressed due to the release from the inhibitory effect of miR-150-5p on the Wnt/β-catenin pathway^44^. This may result in a double-negative regulatory loop where MYC further downregulates hsa-miR-150-5p. In other words, miRNAs can act as either regulators or mediators of MYC function^66^. Altogether, we suggest a new mechanism in which downregulation of tumor suppressive hsa-miR-150-5p, sequestered by hsa-circ-0001955, may contribute to MYC upregulation in CRC patients and its consequences.

### CCNA2

Thus far, Cyclin A2 (CCNA2) has emerged as a multifaceted player in CRC tumor biology, influencing cell-cycle progression, oncogenesis, cell proliferation, and apoptosis. A recent study by Guo et al. demonstrated that CCNA2 is upregulated in almost all primary CRC tumors compared to normal colonic tissues^67^. CCNA2 plays a role in G1/S and G2/M phase transitions by activating CDK1 and CDK2^68^. Additionally, CCNA2 is downstream of the Wnt/β-catenin pathway, which is crucial for cell-cycle progression^69^. A study by Tu et al. showed that FH535, an inhibitor of the Wnt signaling pathway, can inhibit CRC proliferation by downregulating β-catenin, thereby suppressing the Wnt/β-catenin pathway and affecting CCNA2, a downstream proliferation-related gene^69^. Furthermore, Gan et al. revealed that CCNA2 may play an oncogenic role in CRC by regulating cancer cell growth and apoptosis. Its downregulation significantly impairs cell cycle progression and activates apoptosis^70^. Based on these findings, it is likely that the hsa_circ_0001955/hsa-miR-150-5p/CCNA2 axis is involved in cell-cycle progression in CRC. Moreover, CCNA2 can be transcriptionally induced by MYC in mouse mammary glands^71^. Therefore, CCNA2 may be synergistically activated by MYC, which can be induced due to hsa-miR-150-5p downregulation or other mechanisms described for Wnt/β-catenin pathway activation in our paper. The correlation between MYC and CCNA2 is demonstrated in our sub-network. Overall, these findings suggest that the hsa_circ_0001955/hsa-miR-150-5p/CCNA2 axis, along with the involvement of MYC, may play a crucial role in cell-cycle progression and oncogenesis in CRC.

### CD44

CD44 is widely expressed in tumor cells and plays a critical role in various aspects of colorectal cancer (CRC) development and progression, including tumorigenesis, progression, epithelial-mesenchymal transition (EMT), metastasis, and resistance to apoptosis, chemotherapies, and radiotherapies^72–78^. The significant overexpression of CD44 in APC-deficient tumors suggests its key role in intestinal tumorigenesis. Zeilstra et al. reported that CD44 is involved in intestinal tumorigenesis and controls the balance between survival and apoptosis in the intestinal crypt^76^.

CD44 serves as a robust marker of CSCs in CRC and is functionally essential for cancer initiation^79^. Su et al. reported that CD44 could provide stem cell properties for colon cancer cells^80^. Its overexpression intensifies in cells with stem cell properties within tumors, providing them the capability of metastasizing and resistance to therapy. Thus, CD44 overexpression can be used as an indicator of CSCs, and targeting CD44 + cells with therapeutic strategies, such as chemotherapy drugs, can be effective in treating CRC^81^.

In the context of the Wnt/β-catenin pathway, CD44 is a direct target in the intestinal mucosa and is induced by the activation of this pathway. Unlike several other Wnt target genes, CD44 regulation is independent of MYC in the intestinal epithelium^76,82^. Wnt/β-catenin activation in CRC can result from APC mutations and/or may result from downregulation of hsa-miR-150-5p and KLF4, as supported by the literature review and the constructed sub-network. CD44 is not only a Wnt target gene but also a positive regulator of Wnt signaling^83^, and it may play a role in the regulation of the oncogenic YAP signaling pathway. Knockdown of CD44 has been shown to reduce YAP expression^84^, indicating its potential involvement in YAP pathway activation and the upregulation of YAP target genes, such as MYC.

Furthermore, a study in 2016 by Yan et al. reported that CD44 and its variants are negatively regulated by KLF4^85^. This finding is in line with the constructed sub-network, where KLF4 is downregulated through a ceRNA network, potentially leading to the upregulation of CD44. Therefore, KLF4's contribution to CRC stemness may be linked to its interaction with CD44.

Overall, CD44 is a crucial player in CRC development, stemness, and tumorigenicity, and its regulation involves complex interactions with various signaling pathways and transcription factors, including the Wnt/β-catenin pathway and KLF4. Understanding these regulatory mechanisms and their roles in CRC pathogenesis may provide valuable insights for targeted therapeutic approaches in CRC treatment.

### RACGAP1

Rac GTPase activating protein 1, encoded by RACGAP1, functions as a GTPase-activating protein (GAP), and its overexpression is significantly associated with lymph node metastasis and poor prognosis in CRC^86^. An Interesting finding from the study by Yeh et al. is the site dependency of RacGap1’s prognostic effect. Patients with high nuclear RacGAP1 expression showed poor survival rates, while patients with high cytoplasmic RACGAP1 expression exhibited a more favorable prognosis^87^. The hsa_circ_0001955/hsa-miR-150-5p/RACGAP1 axis appears to be a promising area of research that requires more attention. Exploring this axis further may lead to new insights into CRC pathogenesis and provide valuable information for understanding the mechanisms underlying the different prognostic effects of RacGAP1 expression in different cellular compartments. Overall, the role of RACGAP1 and its potential regulatory interactions with hsa_circ_0001955 and hsa-miR-150-5p present an intriguing area of study in CRC research, and further investigations into this axis may help uncover novel aspects of CRC development and progression.

Collectively, these findings yield accurate and reproducible diagnostic and prognostic biomarkers that hold potential clinical applications for predicting the diagnosis and prognosis of CRC. Additionally, they unveil the underlying pathways implicated in CRC development, offering targets for novel treatment approaches. As mentioned earlier, targeting CD44 + cells (an indicator of cancer stem cells) with therapeutic strategies, such as chemotherapy drugs, has proven effective in CRC treatment. Furthermore, the correction of deregulated genes and their malfunction can be achieved by targeting non-coding RNAs within the sub-network. Conversely, other genes and non-coding RNAs contributing to the entire ceRNA network warrant further investigation for their potential roles in CRC tumor biology. It is essential to note, however, that unlike the acceptable number of samples in mRNA and miRNA datasets, the limited number of samples in the circRNA dataset may impact the reproducibility of DECs.

## Conclusion

Based on the information presented in this study, it appears to be a comprehensive investigation that sheds light on the molecular mechanisms underlying colorectal cancer (CRC). The authors have identified novel interactions and cross-talks among circRNAs, miRNAs, and mRNAs including hsa_circ_0071681, and hsa_circ_0001955, hsa-miR-29a-3p, hsa-miR-150-5p, KLF4, MYC, and CD44. These findings hold significant implications for CRC patients, offering potential benefits such as improved diagnosis and targeted therapy. The study suggests that the genes and non-coding RNAs within the network may have a considerable impact on patients' survival rates and could potentially serve as valuable diagnostic and prognostic biomarkers for CRC patients. However, it is crucial to note that this study relies on bioinformatics analyses, and further validation through laboratory studies is necessary to confirm the abnormal expression, functions, and connections of these genes and non-coding RNAs in CRC. Laboratory experiments, such as in vitro and in vivo studies, can provide more concrete evidence and insights into the actual roles of these genes and non-coding RNAs in CRC pathogenesis. Additionally, functional assays can help establish the causal relationships between the identified genes and non-coding RNAs. These assays can shed light on their effects on CRC cell behavior, including proliferation, migration, invasion, and other key processes. Overall, this study establishes a robust foundation for future research in CRC diagnosis, prognosis, and targeted therapy. It proposes potential molecular mechanisms and identifies biomarkers, yet further experimental validation is essential to fully comprehend the biological significance and clinical relevance of these findings.

## Materials and methods

### Data acquisition

For our analysis, we initially selected colorectal cancer-related datasets from the NCBI GEO database (http://www.ncbi.nlm.nih.gov/geo/). In total, seven datasets were chosen for the present study. These included GSE126094 (10 normal tissue samples and 10 tumor tissue samples), GSE115513 (649 normal tissue samples and 853 tumor tissue samples), GSE39582 (19 normal tissue samples and 566 tumor tissue samples) as discovery dataset. Additionally, we included GSE8671 (32 normal tissue samples and 32 tumor tissue samples), GSE9348 (12 normal tissue samples and 70 tumor tissue samples), GSE21510 (25 normal tissue samples and 104 tumor tissue samples), and GSE44076 (148 normal tissue samples and 98 tumor tissue samples) as validation datasets.

### DECs, DEMs, and DEGs analysis

To identify DECs, DEMs, and DEGs in tumor tissues compared to adjacent normal tissues, the following steps were performed. The raw data of GSE126094 were downloaded from the GEO database, and subsequently, background correction and normalization were carried out using the Limma package in R. For the identification of differentially expressed circRNAs (DECs) in GSE126094, the processed data were analyzed using Limma with a cut-off value of an adjusted *p* value of < 0.05 and a |log2 fold change (L2FC)|> 2.0. In the case of GSE115513, quality control of the raw data was performed using the AgiMicroRna package in R. Similar to GSE126094, the processed data of GSE115513 were then analyzed using Limma, applying cut-off values of an adjusted *p* value of < 0.05 and a |log2 fold change (L2FC)|> 0.5 to DEMs. For the identification of DEGs, the quality of raw data in the selected datasets was assessed using the Affy package in R. Background correction and normalization were performed using the Robust Multiarray Average (RMA) function from the Limma package. DEGs were identified using a cut-off value of an adjusted *p* value of < 0.05 and a |log2 fold change (L2FC)|> 1, as determined by Limma.

### Identification of circRNA–miRNA interaction

To identify potential miRNA binding sites on DECs, we utilized the circbank online database (http://www.circbank.cn). The ceRNA hypothesis was then applied to obtain potential target miRNAs for the DECs. We selected the miRNAs that overlapped between the predicted targeting miRNAs of DECs from the circbank tool and DEMs resulting from the GSE115513 analysis. It was a requirement that these selected miRNAs had an expression profile opposite to that of the circRNA that targeted them. DECs that did not target any of the overlapped miRNAs with this condition were excluded from the study.

### Identification of miRNA–mRNA interaction

We utilized the miRWalk online tool (http://mirwalk.umm.uni-heidelberg.de) to identify target genes of the selected miRNAs. The overlapped mRNAs between the predicted targeting mRNAs from the miRWalk database and DEGs resulting from the GSE39582 analysis were chosen for further assessments. It was a requirement that these selected mRNAs exhibited an expression direction opposite to that of the miRNA that targeted them. DEGs that were not targeted by any of the selected miRNAs with this condition were excluded from the study.

### Functional enrichment of selected DEGs

To further insights into the potential function and associated enriched pathways information of selected genes in the ceRNA network, we conducted Gene Ontology (GO) analysis, which included CC, MF, BP analyses, as well as KEGG pathways analysis. These analyses were performed using the ClusterProfiler package in R. Statistical significance was determined using a cut-off value of *p* value < 0.05.

### Construction of circRNA/miRNA/mRNA regulatory network

Based on the ceRNA hypothesis, the obtained DECs/DEMs pairs and DEMs/DEGs pairs were integrated to construct a regulatory network involving circRNAs, miRNAs, and mRNAs. This network was visualized using Cytoscape (version 3.9.1), a powerful software tool for analyzing and visualizing data networks.

### Constructing PPI network and identification of hub genes

Utilizing the online tool STRING, which is dedicated to organism-wide protein association networks, a protein–protein interaction (PPI) network was constructed for the selected DEGs to explore potential interactions. STRING incorporates both known and predicted associations between proteins, encompassing physical and functional associations. A combined score threshold of > 0.4 was applied, and nodes with no connections were removed. The resulting interactions were then imported into Cytoscape for visualization of the PPI network. Subsequently, hub genes were identified using the CytoHubba plugin in Cytoscape software, employing the Maximal Clique Centrality (MCC) ranking method.

### Survival analysis of hub genes

The GEPIA online tool offers a convenient function for obtaining Kaplan–Meier plots of desired genes in various cancer types based on the TCGA database. In our study, we utilized this tool to analyze the association between hub genes and the survival rate COAD, READ, as well as a combined COAD and READ group. To assess the connection between hub genes and overall survival, we employed quartiles as the group cutoff and set a *p* value cutoff of < 0.05 as the threshold for significance. By doing so, we uncovered the prognostic values of the hub genes and selected survival-related hub genes for further analyses.

### Assessment of survival-related hub genes expression profile by validation datasets and TCGA data

In addition to evaluating the expression status of survival-related hub genes in four validation datasets (GSE44076, GSE8671, GSE9348, and GSE21510) obtained from the GEO public repository, we further assessed the expression profiles using the GEPIA online database. GEPIA is a web server that provides tumor/normal differential expression analysis and interactive analyses. For further assessment, we examined the expression profile of survival-related hub genes in the TCGA and the GTEx projects. We compared the COAD and READ groups against matched TCGA normal and GTEx data. A cut-off value of |LogFC|> 1 and p.value < 0.05 were applied following the Log2 transformation (this online tool uses log2 (TPM + 1) for log-scale analysis). Only genes that showed similar expression profiles in all validation datasets and in the GEPIA tool were selected for further assessment.

### ROC curve analysis of hub genes

We utilized the pROC package in R to generate ROC curves, using the expression values obtained from GSE39582. The purpose of these curves was to assess the diagnostic and prognostic potential of the survival-related hub genes in CRC patients. The ROC curves measure the performance of a given test by evaluating the sensitivity (true positives) and specificity (true negatives) at different threshold values. AUC is commonly used to evaluate and select biomarkers. It provides a measure of the overall discriminatory power of the test. In our analysis, we calculated the AUC to assess the performance of the survival-related hub genes as diagnostic and prognostic biomarkers in CRC.

### Supplementary Information

Supplementary Information.

## Data Availability

The authors declare that the data supporting the findings of this study are addressed within the article.
